# Association of Long-term Strenuous Physical Activity and Extensive Sitting With Incident Radiographic Knee Osteoarthritis

**DOI:** 10.1001/jamanetworkopen.2020.4049

**Published:** 2020-05-04

**Authors:** Alison H. Chang, Jungwha (Julia) Lee, Joan S. Chmiel, Orit Almagor, Jing Song, Leena Sharma

**Affiliations:** 1 Department of Physical Therapy and Human Movement Sciences, Northwestern University Feinberg School of Medicine, Chicago, Illinois; 2 Department of Preventive Medicine, Northwestern University Feinberg School of Medicine, Chicago, Illinois; 3 Division of Rheumatology, Department of Medicine, Northwestern University Feinberg School of Medicine, Chicago, Illinois

## Abstract

**Importance:**

Persons with knee symptoms recognize the health benefits of engaging in physical activity, but uncertainty persists about whether regular strenuous physical activity or exercise can accelerate tissue damage. A sedentary lifestyle of inactivity or underloading may also be associated with deleterious joint health.

**Objective:**

To establish whether long-term strenuous physical activity participation and extensive sitting behavior are each associated with increased risk of developing radiographic knee osteoarthritis (KOA) in individuals at high risk for the disease.

**Design, Setting, and Participants:**

This cohort study analyzed data from the Osteoarthritis Initiative, a prospective longitudinal cohort study of men and women with or at an increased risk of developing symptomatic, radiographic KOA. Community-dwelling adults were recruited from 4 US sites (Baltimore, Maryland; Columbus, Ohio; Pittsburgh, Pennsylvania; and Pawtucket, Rhode Island) and were followed up for up to 10 years. Individuals were included if they had a baseline Kellgren and Lawrence grade of 0 in both knees and completed a PASE (Physical Activity Scale for the Elderly) questionnaire at baseline and at least 2 follow-up visits over an 8-year interval. Data analyses were conducted from May 2018 to November 2018.

**Exposures:**

Baseline to 8-year trajectories of strenuous physical activity participation and extensive sitting behavior were identified using group-based trajectory models.

**Main Outcomes and Measures:**

Incident radiographic KOA, defined as Kellgren and Lawrence grade 2 or higher in either knee by the 10-year follow-up visit.

**Results:**

A total of 1194 participants were included in the sample (697 women [58.4%]), with a baseline mean (SD) age of 58.4 (8.9) years and mean body mass index (BMI) of 26.8 (4.5). Four distinct trajectories of weekly hours spent in strenuous physical activities and 3 distinct trajectories of extensive sitting were identified. Long-term engagement in low-to-moderate physical activities (adjusted odds ratio [OR], 0.69; 95% CI, 0.48-1.01) or any strenuous physical activities (adjusted OR, 0.75; 95% CI, 0.53-1.07) was not associated with 10-year incident radiographic KOA. Persistent extensive sitting was not associated with incident KOA. Despite relatively mild symptoms and high function in this early-stage sample, 594 participants (49.7%) did not engage in any strenuous physical activities (ie, 0 h/wk) across 8 years, and 507 (42.5%) engaged in persistent moderate-to-high frequency of extensive sitting. Older age, higher BMI, more severe knee pain, non–college graduate educational level, weaker quadriceps, and depression were each associated with a persistent lack of engagement in strenuous physical activities.

**Conclusions and Relevance:**

Results from this study appeared to show no association between long-term strenuous physical activity participation and incident radiographic KOA. The findings raise the possibility of a protective association between incident KOA and a low-to-moderate level of strenuous physical activities.

## Key Points

**Question:**

Are long-term strenuous physical activity and extensive sitting each associated with risk for developing radiographic knee osteoarthritis in individuals at high risk for the disease?

**Findings:**

In this cohort study of 1194 persons at high risk for but without radiographic evidence of knee osteoarthritis who were followed up for up to 10 years, long-term participation in strenuous physical activities was not associated with risk of incident radiographic knee osteoarthritis. Persistent extensive sitting was not associated with either elevated or reduced risk.

**Meaning:**

These findings suggest that older adults at high risk for knee osteoarthritis may safely engage in strenuous physical activities at a moderate level to improve their general health.

## Teaser

This cohort study evaluates long-term levels of physical activity and inactivity among adults with or likely to develop knee osteoarthritis.

## Introduction

Participation in exercise, sports, or recreational activities provides health benefits,^1^ preserves function,^2^ and prevents disability development.^3,4^ In contrast, sedentary behavior has been associated with adverse health outcomes, independent of physical activity levels.^5,6,7^ Biological mechanisms underlying the advantages of physical activity may be distinct from those associated with deleterious consequences of sedentary behavior. Promoting physical activity and reducing sedentary behavior are low-cost, easy-to-implement strategies to improve health and quality of life.

Although persons with knee symptoms recognize the health benefits of regular physical activity, uncertainty about whether strenuous activity will accelerate joint damage is a common concern.^8,9,10,11,12^ Healthy articular tissues require biomechanical homeostasis.^13^ A lifestyle of inactivity or underloading may also be deleterious to joint health.^14^ The disease-related factors of knee osteoarthritis (KOA), such as pain, buckling, and decreased confidence, often complicate physical activity engagement, especially given that interventions targeting these factors are inadequate. Conversely, persons at high risk but without evidence of radiographic KOA are at a stage when joint symptoms are frequently minimal or mild and tissue damage is not yet widespread. Physical activity programs instituted at this early stage are more likely to be effective and achievable.

Patterns of activity may fluctuate over time, given that the factors associated with them, such as individual health, personal experience, psychosocial attributes, and social and physical environments, are often dynamic.^8,11,12^ Understanding how activity or inactivity levels vary over time and characterizing long-term trajectories could better capture behavior patterns. Examining whether specific physical activities or sedentary trajectories are associated with subsequent KOA disease development and identifying the baseline factors associated with membership in the inferior trajectory subgroups could potentially inform public health messages and guide tailored physical activity promotion and sedentary behavior reduction strategies and interventions.

In this study of a large cohort of community-recruited individuals at high risk for KOA who were followed up for up to 10 years, we aimed to (1) identify the baseline to 8-year trajectories of strenuous physical activity participation and of extensive sitting behavior; (2) evaluate the associations of trajectory subgroup membership for strenuous physical activities and for extensive sitting with incident radiographic KOA during the baseline to 10-year follow-up; and (3) identify baseline factors associated with membership in each of the strenuous physical activity trajectory subgroups and in each of the extensive sitting trajectory subgroups.

## Methods

### Study Sample

The present cohort study is a secondary analysis of the data collected by the Osteoarthritis Initiative (OAI), a prospective longitudinal cohort study of men and women, aged 45 to 79 years at enrollment, with or at an increased risk of developing symptomatic, radiographic KOA.^15^ Annual evaluations of the OAI participants began in 2004 at 4 study sites (Baltimore, Maryland; Columbus, Ohio; Pittsburgh, Pennsylvania; and Pawtucket, Rhode Island). Participants at high risk for KOA but without evidence of radiographic disease (grade 0 under the Kellgren and Lawrence radiographic grading system^16^) in both knees at the baseline visit were included in the present study sample. Eligibility and exclusion criteria for high-risk participants are detailed in eTable in the Supplement. The institutional review board at each site approved the OAI, and all participants gave written informed consent. This present study followed the Strengthening the Reporting of Observational Studies in Epidemiology (STROBE) reporting guideline. Analyses were conducted from May 2018 to November 2018.

### Assessment of Strenuous Physical Activity and Extensive Sitting

Weekly hours of engagement in strenuous physical activities (eg, jogging, swimming, cycling, singles tennis, aerobic dance, and skiing) were estimated over an 8-year follow-up period, using items in the PASE (Physical Activity Scale for the Elderly) questionnaire.^17^ The PASE questionnaire assesses activities in which older adults commonly engaged during the previous week, and it has excellent construct validity^17,18,19,20^ and test-retest reliability.^17,21^ Participants responded to 2 questions. First, they were asked, “Over the past 7 days, how often did you engage in strenuous sport and recreational activities such as jogging, swimming, cycling, singles tennis, aerobic dance, skiing (downhill or cross-country) or other similar activities?” The response options were never, seldom (1-2 days), sometimes (3-4 days), and often (5-7 days). Second, participants were asked, “On average, how many hours/day did you engage in these strenuous sport or recreational activities?,” and were given the following response options: less than 1 hour per day, 1 to fewer than 2 hours per day, 2 to 4 hours per day, and more than 4 hours per day.

Weekly hours of engagement in strenuous physical activities were estimated by combining the midpoint quantity of the frequency and duration responses from the PASE questionnaire. For example, a response of “3-4 days/week” and “1 to <2 hours/day” was estimated as 3.5 days per week and 1.5 hours per day, which produced weekly hours of 5.25.

Extensive sitting behavior was assessed over 8 years, using the sitting items in the PASE questionnaire. Extensive sitting was defined as 5 or more days of sitting activities over the past week and 4 or more hours per day during those days, based on the participant’s responses to 2 questions. First, “Over the past 7 days, how often did you participate in sitting activities, such as reading, watching TV, or doing handicrafts?” Second, “On average, how many hours/day did you engage in these sitting activities?”

### Assessment of Incident Radiographic KOA

Knee radiographs were obtained using the posteroanterior fixed-flexion weight-bearing protocol^22^ with a plexiglass positioning frame (SynaFlexer; Synarc, Inc). The Kellgren and Lawrence grade^16^ was evaluated by 2 OAI experts, who were blind to each other’s reading and all other data. Among participants who showed no evidence of radiographic KOA (Kellgren and Lawrence grade 0) in both knees at baseline, we identified those who developed radiographic KOA (Kellgren and Lawrence grade ≥2) in either knee by the 10-year follow-up visit.

### Assessment of Baseline Demographic and Health-Related Factors

Baseline demographic and health-related variables were selected for analysis based on plausible rationale and/or previous literature concerning physical activity participation and extensive sitting in KOA. Race/ethnicity (white/Caucasian, black/African American, Asian, and other non-white/Caucasian) and educational level (<high school, high school graduate, some college, college graduate, some graduate school, and graduate degree) were self-reported. Body mass index (BMI) was calculated as weight in kilograms divided by height in meters squared.

Comorbidity was assessed with a questionnaire adaptation of the Charlson Comorbidity Index (range: 0-10, with high scores indicating a greater number of comorbidity),^23^ and depressive symptoms were evaluated with the Center for Epidemiologic Studies Depression (CES-D) scale (score range: 0-60, with high scores indicating worse depressive symptoms).^24,25^ Gait speed was measured during a 20-m walk. Knee pain severity was scored with the WOMAC (Western Ontario and McMaster Universities Osteoarthritis Index) Pain subscale (score range: 0-20, with high scores indicating more severe pain), and physical function was scored using the WOMAC Physical Function subscale (score range: 0-68, with high scores indicating worse function),^26^ with each score recorded separately for the right and left knees in the OAI. Isometric knee extensor strength at 60° knee flexion was quantified using the Good Strength Chair.^27^ Knee injury was queried as “Ever injured either knee so badly that it was difficult to walk for at least 1 week?” and knee surgery as “Ever had any kind of knee surgery?” Right and left hip pain was assessed separately with “Have any pain, aching, or stiffness in or around the hip during the past year?” Similar questions were used for right and left ankle and foot pain. The Knee Injury and Osteoarthritis Outcome Score questionnaire^28^ assessed knee confidence and lifestyle modification to avoid potentially knee-damaging activities, with each item scored on a 5-point scale. Self-reported restless sleep during the past week, a CES-D item, was assessed on a 4-point scale.

### Statistical Analysis

First, we identified distinct baseline to 8-year trajectories of strenuous physical activity participation and extensive sitting behavior, using group-based trajectory models in the Stata statistical software traj package (StataCorp LLC).^29^ Individuals who completed baseline and at least 2 follow-up PASE questionnaires were included in the analysis. Using latent mixture modeling with a zero-inflated Poisson distribution, we identified subgroups that shared a similar underlying trajectory of weekly hours spent in strenuous physical activities. To determine the optimal number of trajectory subgroups, we tested models with different numbers of subgroups (2-5) and forms (linear, quadratic, and cubic) of potential trajectories. The final number of subgroups was established according to the bayesian information criterion, trajectory shapes for similarity, and proportion of participants in each subgroup (≥5%).^30^ After identifying the optimal number of groups, we reduced the order of the polynomial for each group until the parameter estimate of the highest degree had a *P* < .05. With the final model, each participant was assigned to 1 of the subgroups on the basis of the maximum posterior predictive probability. We used 4 diagnostic measures to determine trajectory model fit, as suggested by Nagin^31^: (1) the mean posterior probability of assignment for each group was 0.7 or greater, (2) the odds of correct classification were 5.0 or higher, (3) the proportion of a sample assigned to a certain group was close to the proportion estimated from the model, and (4) 99% CIs of the estimated proportion were reasonably narrow. Following a similar approach, we separately identified subgroups of participants with a homogenous underlying trajectory for the probability of extensive sitting behavior.

Second, we evaluated the associations of trajectory subgroup membership for strenuous physical activities (exposure) and for extensive sitting (exposure) with incident radiographic KOA by the 10-year follow-up visit (outcome), using separate person-level logistic regression models unadjusted and adjusted for age, sex, and BMI. Results are reported as odds ratios (ORs) and 95% CIs. We used similar analytic approaches for extensive sitting trajectory subgroups.

Third, we identified baseline factors (exposure) associated with membership in each of the strenuous physical activity trajectory subgroups (outcome) and with membership in each of the extensive sitting trajectory subgroups (outcome), using multinomial logistic regression models, adjusting for baseline factors. Baseline factors included age (continuous); sex (women vs men); educational level (≥college graduate: yes vs no); BMI (continuous); race/ethnicity (white/Caucasian vs non-white/Caucasian); comorbidities (continuous); depressive symptoms (yes [CES-D score ≥16] vs no); gait speed (continuous); WOMAC pain (worse knee) and physical function (worse knee) (continuous); knee extensor strength (worse knee) (continuous); history of knee injury; history of knee surgery; presence of any hip pain, ankle pain, and foot pain; lack of knee confidence and modified lifestyle to avoid knee-damaging activities (yes [score ≥2] vs no); and restless sleep (yes [score ≥2] vs no). Multivariable models were created by including variables with *P* ≤ .20 in the univariate model. Backward selection (removing variables with the adjusted *P* > .20) was used to develop the final models. We summarized the results as adjusted relative risk (RR) ratios and 95% CIs for membership in each of the strenuous physical activity trajectory subgroups. A similar analytic approach was applied to identify baseline factors associated with extensive sitting trajectory subgroup membership.

Likelihood ratio tests and Wald statistics were used to assess statistical significance of the multinomial logistic regression model parameters (RR ratios and 95% CIs). A 95% CI for an RR ratio that excluded the value of 1.0 was considered a statistically significant finding, using a 2-tailed α probability (type 1 error rate) of .05.

## Results

### Strenuous Activities and Extensive Sitting Trajectories

**Figure 1.**
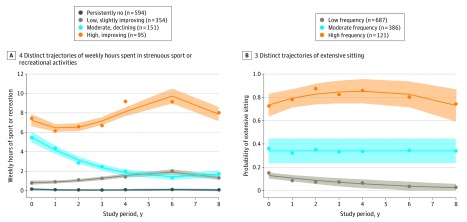
Distinct Trajectories of Weekly Strenuous Physical Activities and Extensive Sitting Over 8 Years Actual (dots) and estimated (solid lines) hours per week (A) and proportion of extensive sitting (B) at each time point and their associated 95% CIs (shaded areas) are shown.

The study sample consisted of 1194 participants (eFigure 1 in the Supplement), of whom 697 were women (58.4%), the mean (SD) age was 58.4 (8.9) years, and the mean (SD) BMI was 26.8 (4.5). We identified 4 distinct trajectories of weekly hours spent in strenuous physical activities (Figure 1A) and 3 distinct extensive sitting trajectories (Figure 1B). For strenuous physical activity, 594 participants (49.7%) were classified into the persistently no subgroup (0 h/wk), and 354 (29.6%) into the low, slightly improving subgroup (1-2 h/wk). Extensive sitting patterns were stable over time. More than 40% were classified into the moderate (386 [32.3%]) to high (121 [10.1%]) frequency of extensive sitting subgroup. Table 1 summarizes baseline demographic and health-related variables for the entire sample, by strenuous physical activities trajectory subgroup, and by extensive sitting trajectory subgroup.

**Table 1.** Baseline Participant Demographic Characteristics and Health-Related Variables

Characteristic	Health-related variable, No. (%)							
	Total (n = 1194)	Weekly strenuous PA trajectory subgroup (n = 1194)				Extensive sitting trajectory subgroup (n = 1194)		
		Persistently no	Low, slightly improving	Moderate, declining	High, improving	High frequency	Moderate frequency	Low frequency
No. (%)		594 (49.7)	354 (29.6)	151 (12.6)	95 (8.0)	121 (10.1)	386 (32.3)	687 (57.5)
Age, mean (SD), y	58.4 (8.9)	59.4 (9.0)	57.2 (8.8)	58.5 (9.2)	56.3 (7.5)	61.1 (8.9)	59.6 (9.2)	57.3 (8.6)
Women	697 (58.4)	357 (60.1)	218 (61.6)	84 (55.6)	38 (40.0)	64 (52.9)	214 (55.4)	419 (61.0)
Race/ethnicity (white/Caucasian)	1014 (84.9)	475 (80.0)	318 (89.8)	134 (88.7)	87 (91.6)	99 (81.8)	319 (82.6)	596 (86.8)
Educational level, ≥college graduate	789 (66.1)	333 (56.1)	257 (72.6)	121 (80.1)	78 (82.1)	71 (58.7)	246 (63.7)	472 (68.7)
BMI, mean (SD)	26.8 (4.5)	27.2 (4.6)	26.6 (4.5)	26.0 (4.3)	25.7 (4.0)	28.0 (5.0)	27.1 (4.6)	26.4 (4.3)
Comorbidities, mean (SD), No.	0.3 (0.8)	0.4 (0.8)	0.3 (0.8)	0.2 (0.6)	0.2 (0.6)	0.6 (1.1)	0.4 (1.0)	0.2 (0.5)
Depressive symptoms, yes^a^	98 (8.2)	66 (11.1)	18 (5.1)	6 (4.0)	8 (8.4)	14 (11.6)	30 (7.8)	54 (7.9)
Gait speed, mean (SD), m/s	1.4 (0.2)	1.3 (0.2)	1.4 (0.2)	1.4 (0.2)	1.4 (0.2)	1.3 (0.2)	1.4 (0.2)	1.4 (0.2)
WOMAC pain, mean (SD)^b^	2.3 (2.9)	2.9 (3.4)	1.8 (2.3)	1.7 (2.0)	1.2 (1.8)	3.0 (3.6)	2.3 (2.8)	2.1 (2.8)
WOMAC function, mean (SD)^c^	6.5 (9.2)	8.4 (10.8)	4.7 (6.9)	4.8 (6.7)	3.6 (6.8)	9.1 (11.6)	6.7 (9.2)	5.9 (8.7)
Knee extensor strength, mean (SD), N	324 (123)	312 (124)	323 (117)	334 (119)	393 (129)	308 (119)	320 (123)	329 (123)
History of knee injury, yes	393 (32.9)	188 (31.6)	110 (31.1)	60 (39.7)	35 (36.8)	46 (38.0)	124 (32.1)	223 (32.5)
History of knee surgery, yes	110 (9.2)	45 (7.6)	32 (9.0)	19 (12.6)	14 (14.7)	9 (7.4)	37 (9.6)	64 (9.3)
Hip pain, yes	285 (23.9)	158 (26.6)	80 (22.6)	31 (20.5)	16 (16.8)	35 (28.9)	99 (25.6)	151 (22.0)
Ankle pain, yes	72 (6.0)	51 (8.6)	15 (4.2)	4 (2.6)	2 (2.1)	8 (6.6)	27 (7.0)	37 (5.4)
Foot pain, yes	89 (7.5)	51 (8.6)	26 (7.3)	8 (5.3)	4 (4.2)	10 (8.3)	28 (7.3)	51 (7.4)
Lack of knee confidence, yes	167 (14.0)	102 (17.2)	39 (11.0)	16 (10.6)	10 (10.5)	24 (19.8)	59 (15.3)	84 (12.2)
Modified lifestyle to avoid knee-damaging PA, yes	244 (20.4)	134 (22.6)	73 (20.6)	22 (14.6)	15 (15.8)	28 (23.1)	87 (22.5)	129 (18.8)
Restless sleep, yes	673 (56.4)	349 (58.8)	186 (52.5)	85 (56.3)	53 (55.8)	81 (66.9)	211 (54.7)	381 (55.5)

Abbreviations: BMI, body mass index (calculated as weight in kilograms divided by height in meters squared); N, newton; PA, physical activity; WOMAC, Western Ontario and McMaster Universities Osteoarthritis Index.

^a^ Center for Epidemiologic Studies Depression scale score 16 or greater.

^b^ WOMAC pain subscale score range: 0-20, with high scores indicating more severe pain.

^c^ WOMAC function subscale score range: 0-68, with high scores indicating worse function.

### Associations of Trajectory Subgroup With Incident Radiographic KOA

**Figure 2.**
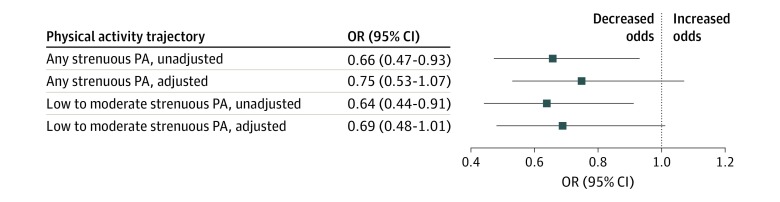
Associations of Weekly Strenuous Physical Activity Trajectories With 10-Year Incident Radiographic Knee Osteoarthritis The reference group is persistently no strenuous physical activity (PA) over 8 years. A 95% CI below the value of 1.0 supports a statistically significant reduced likelihood of incident knee osteoarthritis. Adjusted indicates adjusted for age, sex, and body mass index; OR, odds ratio; unadjusted, unadjusted odds.

Among 1194 participants, 155 (13.0%) developed radiographic KOA by the 10-year follow-up visit. The respective radiographic KOA incidence rates for the 4 strenuous physical activities trajectory subgroups were 15.3% (persistently no), 10.7% (low, slightly improving), 9.3% (moderate, declining), and 12.6% (high, improving). For the 3 extensive sitting trajectory subgroups, the incidence rates were 14.0% (high frequency), 13.7% (moderate frequency), and 12.4% (low frequency). Compared with participating in persistently no strenuous physical activities, participating in any (unadjusted OR, 0.66; 95% CI, 0.47-0.93) and in low-to-moderate level strenuous physical activities (unadjusted OR, 0.64; 95% CI, 0.44-0.91) over 8 years were each associated with a reduced likelihood of incident radiographic KOA over the baseline to 10-year follow-up period (Figure 2). After adjusting for age, sex, and BMI, we found the ORs to be similar but not statistically significant for any (adjusted OR, 0.75; 95% CI, 0.53-1.07) and for low-to-moderate level strenuous physical activities (adjusted OR, 0.69; 95% CI, 0.48-1.01) (Figure 2). Participating in high (vs persistently no) strenuous physical activities was not associated with incident KOA (unadjusted OR, 0.80; 95% CI, 0.42-1.52; adjusted OR, 1.07; 95% CI, 0.55-2.09). Further adjusting for extensive sitting trajectory minimally altered the ORs. We found no statistical evidence of an association between extensive sitting trajectory and incident KOA (eFigure 2 in the Supplement).

### Associations of Baseline Factors With Trajectory Subgroup

As shown in Table 2, being older (RR ratio per 5-year increase, 0.85; 95% CI, 0.73-0.99), having higher BMI (RR ratio per 5-unit increase, 0.66; 95% CI, 0.48-0.90), and having more severe knee pain (RR ratio per 3-unit increase, 0.58; 95% CI, 0.40-0.84) were each associated with a reduced likelihood of membership in the high, improving strenuous physical activities subgroup. Being a college graduate (RR ratio, 1.90; 95% CI, 1.06-3.41) and having stronger quadriceps (RR ratio per 60-N increase, 1.27; 95% CI, 1.13-1.43) were each associated with an increased likelihood of membership in the high, improving subgroup (reference subgroup: persistently no). Having depressive symptoms was associated with a reduced likelihood of membership in the moderate, declining (RR ratio, 0.38; 95% CI, 0.16-0.92) or low, slightly improving (RR ratio, 0.49; 95% CI, 0.28-0.88) subgroup.

**Table 2.** Association of Baseline Factors With Membership in Each Weekly Strenuous Physical Activities Trajectory Subgroup

Baseline factor^a^	RR ratio (95% CI)^b^		
	Weekly strenuous PA trajectory subgroup		
	Low, slightly improving (n = 354)	Moderate, declining (n = 151)	High, improving (n = 95)
Age, per 5-y increase	0.84 (0.77-0.92)^c^	0.90 (0.81-1.01)	0.85 (0.73-0.99)^c^
BMI, per 5-unit increase	0.94 (0.80-1.11)	0.75 (0.60-0.95)^c^	0.66 (0.48-0.90)^c^
White/Caucasian race/ethnicity, reference: non-white	1.78 (1.14-2.78)^c^	1.15 (0.64-2.07)	1.23 (0.55-2.74)
Depressive symptoms, yes vs no^d^	0.49 (0.28-0.88)^c^	0.38 (0.16-0.92)^c^	0.83 (0.36-1.92)
WOMAC pain, per 3-unit increase^e^	0.74 (0.62-0.88)^c^	0.68 (0.53-0.88)^c^	0.58 (0.40-0.84)^c^
Knee extensor strength, per 60 N increase^f^	0.94 (0.89-1.06)	1.06 (0.94-1.13)	1.27 (1.13-1.43)^c^
Educational level, ≥college graduate vs ≤ some college	1.68 (1.23-2.29)^c^	2.51 (1.58-3.98)^c^	1.90 (1.06-3.41)^c^

Abbreviations: BMI, body mass index (calculated as weight in kilograms divided by height in meters squared); N, newton; PA, physical activity; RR, relative risk; WOMAC, Western Ontario and McMaster Universities Osteoarthritis Index.

^a^ Baseline factors in the final models included those with *P* ≤ .20 in the initial univariate models and then in the multivariable models.

^b^ Multinomial logistic regression model results (n = 1194): RR ratio greater than 1 indicates increased likelihood of being in the better trajectory subgroups (eg, more strenuous PA); RR ratio less than 1 indicates reduced likelihood (reference group: persistently no strenuous PA, which is an inferior trajectory).

^c^ 95% CI that excludes 1 indicates statistical significance.

^d^ Center for Epidemiologic Studies Depression scale score ≥16.

^e^ WOMAC pain subscale score range: 0-20, with high scores indicating more severe pain.

^f^ Strength range: 27 N-880 N.

As shown in Table 3, being older (RR ratio per 5-year increase, 1.25; 95% CI, 1.11-1.42), having higher BMI (RR ratio per 5-unit increase, 1.47; 95% CI, 1.16-1.86), and having more comorbidities (RR ratio, 1.36; 95% CI, 1.08-1.71) were each associated with an increased likelihood of membership in the high frequency of extensive sitting (reference subgroup: low frequency). Similar statistically significant associations were observed in the moderate frequency subgroup, with slightly smaller RR ratios (older age: RR ratio, 1.14 [95% CI, 1.05-1.23]; higher BMI: RR ratio, 1.18 [95% CI, 1.01-1.37]; more comorbidities: RR ratio, 1.28 [95% CI, 1.07-1.54]).

**Table 3.** Association of Baseline Factors With Membership in Each Extensive Sitting Trajectory Subgroup

Baseline factor^a^	RR ratio (95% CI)^b^	
	Extensive sitting trajectory subgroup	
	Moderate frequency (n = 386)	High frequency (n = 121)
Age, per 5-y increase	1.14 (1.05-1.23)^c^	1.25 (1.11-1.42)^c^
Women, reference: men	0.68 (0.49-0.95)^c^	0.63 (0.38-1.05)
BMI, per 5-unit increase	1.18 (1.01-1.37)^c^	1.47 (1.16-1.86)^c^
Depressive symptoms, yes vs no^d^	1.05 (0.65-1.71)	1.45 (0.73-2.90)
Comorbidity, per 1-unit increase^e^	1.28 (1.07-1.54)^c^	1.36 (1.08-1.71)^c^
WOMAC pain, per 3-unit increase^f^	1.02 (0.88-1.18)	1.22 (1.00-1.50)
Knee extensor strength, per 60 N increase^g^	0.94 (0.84-1.00)	0.89 (0.79-1.00)

Abbreviations: BMI, body mass index (calculated as weight in kilograms divided by height in meters squared); N, newton; RR, relative risk; WOMAC, Western Ontario and McMaster Universities Osteoarthritis Index.

^a^ Baseline factors in the final models included those with *P* ≤ .20 in the initial univariate models and then in the multivariable models.

^b^ Multinomial logistic regression model results (n = 1194): RR ratio greater than 1 indicates increased likelihood of being in the inferior trajectory subgroup (eg, moderate and high frequency of extensive sitting); RR ratio less than 1 indicates reduced likelihood (reference group: low frequency of extensive sitting, which is a good trajectory).

^c^ 95% CI that excludes 1 indicates statistical significance.

^d^ Center for Epidemiologic Studies Depression scale score ≥16.

^e^ Comorbidity score range: 0-10, with a higher score indicating a greater number of comorbidities.

^f^ WOMAC pain subscale score range: 0-20, with high scores indicating more severe pain.

^g^ Strength range: 27-880 N.

## Discussion

In persons at high risk but without radiographic evidence of KOA, engaging in long-term strenuous physical activity (eg, jogging, swimming, cycling, singles tennis, aerobic dance, and skiing) over an 8-year period was not associated with risk of incident radiographic KOA. The adjusted ORs ranged from 0.69 (95% CI, 0.48-1.01) for low-to-moderate level of strenuous physical activities to 0.75 (95% CI 0.53-1.07) for any strenuous physical activities, suggesting that, even in persons at high risk for KOA, strenuous physical activity is not associated with development of radiographic changes. These findings are consistent with the possibility that some level of strenuous physical activity, especially in low and moderate amounts (eg, 1-2 hours/wk), may be protective. We believe that these results also convey a reassuring message that older adults at high risk for KOA for whom regular physical activity provides multiple health benefits may safely engage in long-term strenuous physical activity at a moderate level. Extensive sitting trajectories were not associated with either elevated or reduced risk of incident radiographic KOA. To our knowledge, this study is the first to characterize physical activity and sedentary behavior patterns over an extended interval and to assess their associations with incident KOA in persons at high risk for KOA.

The advisory committee of the 2018 Physical Activity Guidelines for Americans emphasized the confusion about whether specific physical activity amounts and intensities were associated with KOA disease worsening.^32^ Persons with knee symptoms often avoid or curtail vigorous physical activity for fear of further harm.^8,9,10,11,12^ Health care practitioners also feel that they have inadequate evidence to unequivocally reassure patients concerning intense physical activity.^33^ Physical activity and sedentary measures at a single time point may not capture an individual’s average habitual activity and/or behavior over an extended period.

We identified 4 distinct trajectories of weekly hours of strenuous physical activities (persistently no; low, slightly improving; moderate, declining; and high, improving) and 3 distinct stable extensive sitting trajectories (high, moderate, and low frequencies) over an 8-year interval. Despite relatively mild symptoms and high function, nearly 50% of participants did not engage in any strenuous physical activities and approximately 30% engaged in activities for 1 to 2 hours per week across 8 years. More than 40% of participants were classified into moderate to high frequency of extensive sitting subgroups. These findings are in agreement with those from other longitudinal studies of community-dwelling older adults that are not limited to persons at high risk for KOA (follow-up: 8 to 12 years).^34,35,36,37^ Most of these studies identified 3 or 4 distinct physical activity trajectory subgroups,^38^ and most participants (32%-75%)^34,35,36,37^ were classified into the consistently inactive subgroup.

Sedentary behavior has emerged in recent years as an independent factor for poor health and function. In a conference report of 878 persons with symptomatic knee and/or hip osteoarthritis followed up for 5 years, Bitar and colleagues^39^ identified 3 stable trajectories of sedentary behavior; 43% of the study sample were in the consistently moderate or high sedentary behavior subgroups. Findings of the present study suggest that lack of strenuous physical activity participation and frequent extensive sitting are prevalent and appear to be entrenched, even in those at high risk for but not yet diagnosed with radiographic disease. These observations underscore the need to incorporate physical activity counseling as part of the standard care for high-risk individuals, at an early stage when physical activity engagement is more attainable.

Exercise and physical activity are recommended as first-line management for KOA.^40,41^ Limited efficacy of current management may in part be attributable to its implementation at a later disease stage, when structural damage already exists^42^ and chronic pain sensitization may have occurred.^43^ The early stage for the present sample represented an opportunity to proactively intervene for optimal therapeutic outcome. As shown in Table 2, compared with the persistently no strenuous physical activity subgroup, the other 3 subgroups shared a similar profile of RR magnitude and direction. Our findings suggest that targeting modifiable factors, including BMI, knee pain, depression, and quadriceps strength, may increase long-term any physical activity participation. In addition, physical activity promotion and sedentary reduction efforts may incorporate tailored approaches to engage those who are likely to be persistently inactive because of age, comorbidities, or educational level.

### Strengths and Limitations

This study has several strengths. First, it analyzed data from the OAI, a large, well-characterized, comprehensively assessed, multiple community–recruited cohort followed up over an extended period. Second, it focused on persons at high risk for KOA, for whom increasing physical activity was more likely to be successful and achievable before the onset of joint damage and chronic pain. Third, it applied group-based trajectory models of activity and inactivity as dynamic behaviors that may vary over time.

This study also has limitations. We used self-reported measures to assess physical activity participation and sedentary behavior, which could be subject to reporting biases.^44^ However, using accelerometry and processing minute-by-minute recordings to quantify activity or inactivity over 8 years in more than 1000 participants would be exceedingly costly, time consuming, and burdensome to participants. Only 13.0% of the sample developed radiographic KOA by the 10-year follow-up visit. Longer follow-up time with more incident cases will further elucidate the association between physical activity patterns over time and disease development. These findings are more generalizable to those at high risk for KOA with similar age and educational levels. We cannot generalize the findings to populations without these characteristics. Despite including plausible factors and adjusting for confounders, unmeasured residual confounding is still possible.

## Conclusions

This cohort study found that, among 1194 persons at high risk for KOA followed up for up to 10 years, long-term strenuous physical activity participation was not associated with increased odds of incident radiographic KOA. Findings suggest that older adults at high risk for KOA may safely engage in strenuous physical activity at a moderate level to promote their general health and well-being. We identified 4 distinct trajectories of strenuous physical activities and 3 distinct stable trajectories of extensive sitting. In this cohort with relatively high function and mild pain, nearly 50% of the participants did not engage in any strenuous physical activity and more than 40% had moderate to high frequency of extensive sitting.

## Notes

Supplement. **eTable.** Eligibility and Exclusion Criteria for Participants at High Risk for Knee Osteoarthritis **eFigure 1.** Derivation of Analysis Sample (n = 1194) **eFigure 2.** Associations of Extensive Sitting Trajectories With 10-Year Incident Radiographic Knee Osteoarthritis
