# Supplementary material for: Association of Long-term Strenuous Physical Activity and Extensive Sitting With Incident Radiographic Knee Osteoarthritis
Source: JAMA Netw Open. 2020 May 4;3(5):e204049. doi: 10.1001/jamanetworkopen.2020.4049 (PMC7199114; doi:10.1001/jamanetworkopen.2020.4049)
Supplement: Supplement. — eTable. Eligibility and Exclusion Criteria for Participants at High Risk for Knee Osteoarthritis eFigure 1. Derivation of Analysis Sample (n = 1194) eFigure 2. Associations of Extensive Sitting Trajectories With 10-Year Incident Radiographic Knee Osteoarthritis [file jamanetwopen-3-e204049-s001.pdf]

Supplementary Online Content

Chang AH, Lee J, Chmiel JS, Almagor O, Song J, Sharma L. Association of long-term strenuous physical activity and extensive sitting with incident radiographic knee osteoarthritis. *JAMA Netw Open.* 2020;3(5):e204049. doi:10.1001/jamanetworkopen.2020.4049

eTable. Eligibility and Exclusion Criteria for Participants at High Risk for Knee Osteoarthritis

eFigure 1. Derivation of Analysis Sample (n = 1194)

eFigure 2. Associations of Extensive Sitting Trajectories With 10-Year Incident Radiographic Knee Osteoarthritis

This supplementary material has been provided by the authors to give readers additional information about their work.

©2020 Chang AH et al. *JAMA Network Open*

eTable. Eligibility and Exclusion Criteria for Participants at High Risk for Knee Osteoarthritis

**Criteria**	**Description**
Age and sex distribution	Proportional to the US population
Racial/ethnic minority representation	Per representation in respective area
Enrollment site	Baltimore, Maryland; Columbus, Ohio; Pittsburgh, Pennsylvania; or Pawtucket, Rhode Island
Eligibility	One or more of the following, in the absence of radiographic knee osteoarthritis:
	Frequent knee symptoms (most days of 1 mo in the past 12 mo)
	Excess body weight (body weight above the median for each age- and sex-specific group, based on data from the Framingham Osteoarthritis Study)
	Prior knee injury causing walking difficulty for at least 1 wk
	Previous knee surgery
	Family history of knee replacement
	Heberden nodes
	Repetitive knee bending
	Aged 70-79 y
Exclusion	Inflammatory arthritis
	Severe bilateral joint space narrowing
	Knee replacement and severe contralateral narrowing
	Bilateral knee replacement or plan for it within 3 y
	Magnetic resonance imaging contraindications
	Use of aids for >50% of ambulation (except 1 cane)
	Severe comorbidity
	Double-blind clinical trial participation
Recruitment	Community-dwelling individuals recruited through population lists, mass mailing of study materials, and media and community outreach

©2020 Chang AH et al. *JAMA Network Open*

eFigure 1. Derivation of Analysis Sample (n = 1194)

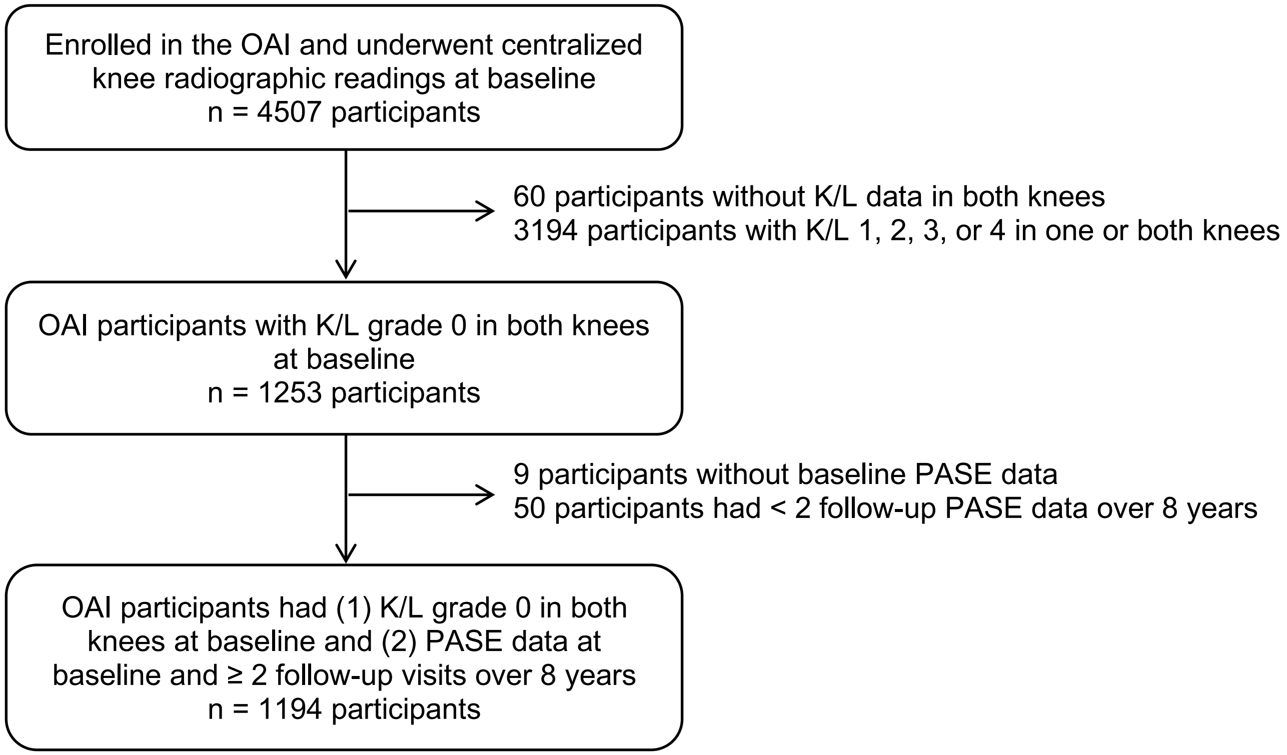

OAI: Osteoarthritis Initiative; K/L: Kellgren/Lawrence; PASE: Physical Activity Scale for the Elderly

©2020 Chang AH et al. *JAMA Network Open*

eFigure 2. Associations of Extensive Sitting Trajectories With 10-Year Incident Radiographic Knee Osteoarthritis

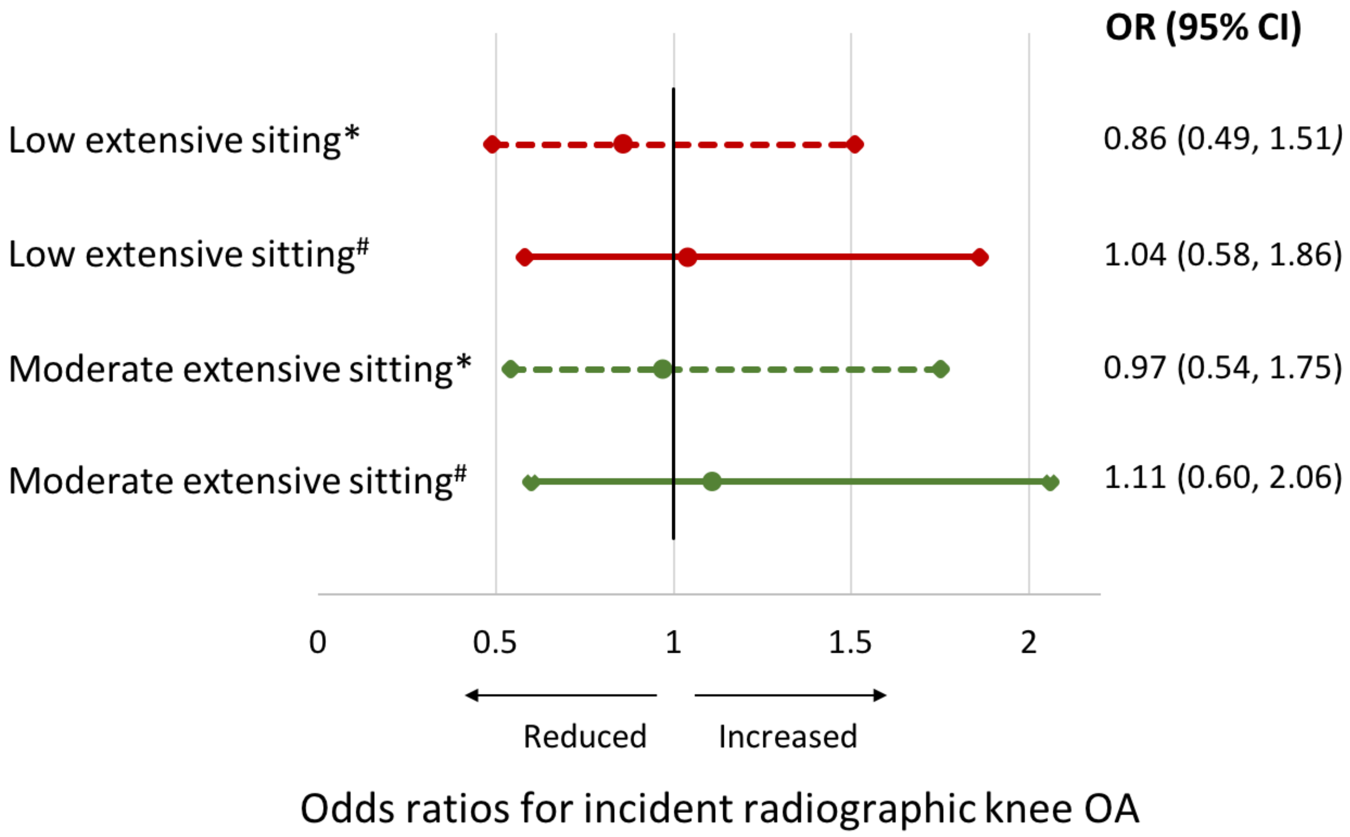

Red marks and lines represent low extensive sitting trajectory subgroup over 8 years. Green marks and lines represent moderate extensive sitting trajectory subgroup. Reference group: high extensive sitting trajectory subgroup. Odds ratios (ORs) and 95% confidence intervals (CIs) are plotted; a 95% CI that is completely below the value of 1 supports a statistically significant reduced likelihood of incident knee osteoarthritis.
*unadjusted (dashed lines); #adjusted for age, sex, and BMI (solid lines)

©2020 Chang AH et al. *JAMA Network Open*
